# Variation in the extent to which patient information leaflets describe potential benefits and harms of trial interventions: a commentary

**DOI:** 10.1186/s13063-025-08824-8

**Published:** 2025-04-14

**Authors:** Jeremy Howick, John D. Lantos, Shaun Treweek, Martina Svobodova, Nina Jacob, Adrian Edwards, Jennifer Bostock, Peter Bower, Katie Gillies, Kerenza Hood

**Affiliations:** 1 Cardiff University Centre for Trials Research, Cardiff University, Cardiff, Wales UK; 2 Stoneygate Centre for Empathic Healthcare, University of Leicester, George Davies Centre, Lancaster Road, Leicester, LE1 7HA UK; 3 Presbyterian Hospital, New York, NY USA; 4 Aberdeen Centre for Evaluation, University of Aberdeen, Aberdeen, Scotland UK; 5 Division of Population Medicine, Cardiff University, Cardiff, Wales UK; 6 Independent, Oxford, UK; 7 Centre for Primary Care, Manchester University, Manchester, UK

**Keywords:** Harms, Benefits, Clinical trials, Informed consent, Autonomy, Risks, Non-maleficence, Beneficence, Nocebo

## Abstract

Clinical trial participants must understand the possible risks and benefits of trial interventions before providing their informed consent to participate. The aim of this commentary is twofold: to summarize the discrepancies in the extent to which patient information leaflets (PILs) list potential benefits and harms of trial interventions; and to highlight subsequent ethical issues that may result from failure to disclose potential benefits or harms . A review of 247 patient information leaflets (PILs) found that the extent to which potential benefits and harms are described varies, with 28 (11%) not describing potential benefits and 23 (9%) not describing potential harms. We argue that there is no principled difference between potential benefits and potential harms (what is helpful for one person could harm another), and the need to disclose potential benefits may be less accepted than the need to disclose all potential harms. Additionally, while it is recognized that failure to mention potential harms may violate the ethical principle of autonomy, it is less well-established that other ethical principles, (the need to avoid harm (non-maleficence) , to help patients (beneficence), and to promote justice) may also be at risk when all potential harms and benefits are not  disclosed within PILs. We suggest that the way potential benefits and harms are described within PILs be harmonized according to recently established principles.

## Background

Study participants must be informed of the potential risks as well as the potential benefits of trial interventions [1–3]. Hence, the Declaration of Helsinki states: “each potential participant must be adequately informed in plain language of … the anticipated benefits and potential risks and burdens... of the research” [3]. This is also what participants want. A recent study asking patient representatives and research ethic committee members (“institutional review boards” in the USA) generated clear consensus that informing trial participants of both potential trial harms as well as potential benefits was desired [4, 5].

Despite the need to mention potential benefits and harms, the extent to which potential benefits are shared with trial participants varies. A review of 33 PILs in the UK found that 3 (9%) did not describe potential harms, and 10 (30%) did not describe potential benefits (see Table 1) [6]. In a larger sample of 214 PILs, 20 (9%) did not disclose potential harms, and 18 (8%) did not disclose potential benefits [7].

**Table 1:** Disclosure of potential benefits and potential harms in two samples of PILs

**Study**	**Total number of PILs in sample**	***n*** ** (%) that did not disclose benefits**^**a**^	***n*** ** (%) that did not disclose harms**^**a**^
Kirby 2020	33	10 (30%)	3 (9%)
Cuddihy 2024	214	18 (8%)	20 (9%)
Total	247	28 (11%)	23 (9%)

^a^This refers to unique PILs

## On the need to disclose potential benefits and harms

Well-publicized studies such as the Tuskegee Syphilis Study [8] and the Human Radiation Experiments [9] in which people were harmed as a result of not being informed that they were participating in a trial, let alone the risks of the trial, illustrate the dangers of doing research without full disclosure of the risks. While some disagree over correct terminology (“side effect,” “harm,” “safety incident”) [10], withholding knowledge of potential harms, however described, is unlikely to be justifiable given the requirement to disclose information.

Possible exceptions to this rule have been proposed. In one example, it was recommended that potential participants be asked how much they would like to know about potential benefits or harms [11]. If participants consent to remaining uninformed, withholding information might seem acceptable. However, this approach is flawed because participants cannot evaluate what information is being withheld, rendering their choice to remain ignorant essentially uninformed. At best, such a recommendation might apply to the depth of discussion with a clinician about potential harms, but it does not justify omitting critical information about potential harms from the participant information leaflet (PIL).

Others have claimed that it is acceptable to withhold information about potential harms if doing so benefits the participant [12]. For example, informing the participant about some mild harms could lead the participant to expect the harm, and for some types of outcomes (such as pain) the expectation of the harm could even cause the harm (a “nocebo” effect) [13]. However, even if withholding information reduces the risk of some harm, it must be established that the reduced harm outweighs the actual (moral) harm of withholding information. Additionally, there are different ways to explain potential harms, some of which are less likely to cause nocebo effects than others [14]. Hence, it may not be necessary to withhold information about harms to avoid causing nocebo effects. It could also arguably be acceptable to withhold information about potential harms if the intervention is very unlikely to have any adverse effects. For example, one of the PILs within our sample that did not provide any information about possible adverse events was for a trial of reminding school children to brush their teeth [15]. The PIL for this trial did not mention harms, which seems acceptable. However, some of the PILs within our sample that did not mention harms arguably should have. One of these was for a placebo-controlled trial of eculizumab to treat children with a blood disorder called Shiga toxin-producing *Escherichia coli* hemolytic uremic syndrome (STEC-HUS) [16, 17]. The PIL did not state any of the potential harms following eculizumab treatment, whereas adverse events related to eculizumab for STEC-HUS include neurological impairment and death [18]. (Aside: the trial also used a placebo control whereas there are other available treatments for STEC-HUS such as volume expansion [19]. The use of the placebo control without the inclusion of other potentially helpful interventions is an additional problem [14]).

 Failure to mention potential benefits is also problematic. For example, some PILs within our sample stated that there were no potential benefits of the placebo control intervention. This is not the case. While there is a debate about how effective placebo treatments are [20], there is little doubt that they have some effects, including non-specific effects that apply across conditions such as reducing anxiety and pain [14].

## A note on the mistaken distinction between potential benefits and potential harms

In December 2020, we participated in a workshop with representatives from the Health Research Authority (HRA), ethics committee members, and trial managers (total 25 participants) [21]. The purpose of the workshop was to co-produce recommendations for developing guidance and resources related to attempts to harmonize the way potential benefits and harms are shared within PILs. Some of the workshop participants expressed a belief that research regulations prohibited discussion of the potential benefits of participating in a study within PILs. The justifications given were that whereas potential harms were allegedly known, potential benefits were not, and that mentioning potential benefits was coercive and possibly illegal. The belief that potential benefits are less certain than potential harms is mistaken. The extent to which potential benefits or harms are known depends on the state of background evidence, which in the case of drugs is related to the phase of the trial (see below). Moreover, there is no fundamental distinction between potential benefits and potential harms, because whether something counts as a benefit or harm is often relative to the individual. For example, a side effect of selective serotonin reuptake inhibitors is sexual dysfunction [22]. This is undesirable to many, but is a benefit to benefit for people with premature ejaculation [22]. Likewise, loss of appetite could be a negative effect of a drug for some but may be judged a positive effect by those who are trying to lose weight. In a more extreme example, morphine slows down bodily functions (considered a harm to many) yet is a benefit to those who are deeply suffering and happy for their functions to slow down painlessly even if it increases the risk of their demise. Hence, mentioning potential risks without mentioning potential benefits is often logically impossible. Regarding whether mentioning potential benefits is coercive, we disagree. While exaggerating potential benefits could be coercive, listing them is not. In fact, withholding information about potential benefits could amount to coercing participants into opting out of trials that could benefit them.

## The extent to which potential benefits and harms should be listed depends on the type of trial

Taking drug trials as an exemplar, despite having strong pathophysiologic rationales and data from observational [23] and mechanistic studies, many drugs tested in phase I safety trials fail to translate to the clinical setting [24, 25]. Sometimes, serious unexpected harms are also discovered [26]. Therefore, it is rarely appropriate to emphasize potential benefits at this stage, and it is important to emphasize the possibility of unexpected (potentially serious) harms. However, once drugs demonstrate likely safety in phase I trials and progress to phase II efficacy studies, if some efficacy has been observed in phase I, it becomes increasingly legitimate to mention both potential benefits and harms. Here, claims about potential benefits must remain very tentative because about one in three drugs tested in phase II trials show no efficacy (or are revealed as harmful) and are abandoned [27]. Once the intervention confirms safety and demonstrates some potential benefit in phase II and proceeds to a phase III trial, then it is legitimate to mention both potential benefits and potential harms because both will be evidence-based. Similar principles apply to non-drug trials such as surgical and intervention trials [28], where the certainty with which potential benefits and harms can be claimed increases as the evidence becomes more voluminous and rigorous.

## Ethics of failure to mention potential benefits or harms

Autonomy may be compromised by omitting information about potential benefits or harms since withholding this information prevents people making an informed choice [5]. Hence, the World Health Organization’s Declaration of Helsinki [3] as well as requirements for informed consent in the UK [29], USA [30], and the European Union [31], all mention the need to disclose all information about both potential harms and potential benefits. It is less well recognised that other ethical principles may be violated when information about potential benefits or harms is not mentioned. Non-maleficence (the requirement to avoid harm) could be violated if potential benefits are not mentioned since this leads to an unbalanced, over-emphasis on potential harms that increases risk of harmful nocebo effects [6, 32]. Relatedly, people who are scared away from participating in a trial because of failure to mention potential benefits are foregoing an opportunity to gain from the trial, which violates beneficence (the ethical requirement to help whenever possible). Relatedly, again if participants are scared away from a trial because potential benefits are not mentioned alongside potential harms, justice (including fair distribution of resources) could be violated. This is because clinical trials often fail because of difficulties in recruiting and/or retaining patients [33]. Ensuring that potential benefits are described alongside potential risks could also improve recruitment and retention rates [34], thus improving justice by wasting less money on trials that failed to recruit or retain patients. 

## Solution: describe all potential benefits and potential harms in close proximity

A survey of 250 stakeholders (including patient and public representatives, research ethics committee members, industry representatives, medico-legal experts, psychologists, and trial managers) reached a consensus about seven principles that can be used to harmonize the way potential benefits and harms are shared with participants in trials. These were (1) all potential harms of a given intervention should be listed, (2) all potential harms should be separated into serious and less serious, (3) it must be made explicit that not all potential harms are known, (4) all potential benefits should be listed, (5) all potential benefits and harms need to be compared with what would happen if the participant did not take part in the trial, (6) suitable visual representations should be added where appropriate, and (7) information regarding potential benefits and harms should not be presented apart by one or more pages [5]. Adhering to these principles would ensure inclusion of all known potential benefits and harms within PILs in a way that potential trial participants could straightforwardly compare them, thus potentially improving the ethics of PILs and clinical trials.

## Conclusion

Every intervention has potential effects, and these effects will be beneficial to some and harmful to others. Deciding whether to enroll in a trial (or take a medication) relies on weighing up these potential benefits and harms. It is impossible to do this if either the benefits or harms are not mentioned, and it is difficult to do so if the potential benefits and harms are not mentioned in close textual proximity. Trial participants need to be informed of these effects in a way they find acceptable, and there is now an established acceptable method to achieve this which should now be followed.

## Data Availability

There are no additional data and materials associated with this manuscript.

## Abbreviations

AIDS: Acquired immune deficiency syndrome
CER: Comparative effectiveness research
EU: European Union
HIV: Human immunodeficiency viruses
IRB: Institutional review boards
OHRP: Office of Human Research Protections
PIL: Patient information leaflet
REB: Research ethics board
